# Efficient Targeted Mutagenesis in Apple and First Time Edition of Pear Using the CRISPR-Cas9 System

**DOI:** 10.3389/fpls.2019.00040

**Published:** 2019-02-06

**Authors:** Aurélie Charrier, Emilie Vergne, Nicolas Dousset, Andréa Richer, Aurélien Petiteau, Elisabeth Chevreau

**Affiliations:** IRHS, INRA, Agrocampus-Ouest, Université d’Angers, SFR 4207 QuaSaV, Beaucouzé, France

**Keywords:** apple, pear, gene editing, knock-out, CRISPR, PDS, TFL1

## Abstract

Targeted genome engineering has emerged as an alternative to classical plant breeding and transgenic methods to improve crop plants. Among other methods (zinc finger nucleases or TAL effector nucleases) the CRISPR-Cas system proved to be the most effective, convenient and least expensive method. In this study, we optimized the conditions of application of this system on apple and explored its feasibility on pear. As a proof of concept, we chose to knock-out the Phytoene Desaturase (*PDS*) and Terminal Flower 1 (*TFL1*) genes. To improve the edition efficiency, two different single guide RNAs (gRNAs) were associated to the Cas9 nuclease for each target gene. These gRNAs were placed under the control of the U3 and U6 apple promoters. Characteristic albino phenotype was obtained for 85% of the apple transgenic lines targeted in *MdPDS* gene. Early flowering was observed in 93% of the apple transgenic lines targeted in *MdTFL1.1* gene and 9% of the pear transgenic lines targeted in *PcTFL1.1*. Sequencing of the target zones in apple and pear CRISPR-PDS and CRISPR-TFL1.1 transgenic lines showed that the two gRNAs induced mutations but at variable frequencies. In most cases, Cas9 nuclease cut the DNA in the twenty targeted base pairs near the protospacer adjacent motif and insertions were more frequent than deletions or substitutions. The most frequent edition profile of *PDS* as well as *TFL1.1* genes was chimeric biallelic. Analysis of a sample of potential off-target sequences of the CRISPR-TFL1.1 construct indicated the absence of edition in cases of three mismatches. In addition, transient transformation with the CRISPR-PDS construct produced two T-DNA free edited apple lines. Our overall results indicate that, despite the frequent occurrence of chimerism, the CRISPR-Cas 9 system is a powerful and precise method to induce targeted mutagenesis in the first generation of apple and pear transgenic lines.

## Introduction

Apple (*Malus* x *domestica* Bork.) is one of the major fruit crops produced in the world with a production over 89 million tons in 2016. The world pear production in 2016 reached 27 millions tons, including both European pears (*Pyrus communis* L.) and Asian pears (P. sp.) (FAOSTAT^1^). Conventional breeding of both species is limited by their long reproductive cycle and their high degree of heterozygosity. In addition, most fruit trees are produced by clonal propagation, traditional cultivars are still dominant and the speed of introduction of new hybrid varieties on the market is slow. In this context, genetic engineering appears as a powerful tool to accelerate the improvement of existing apple and pear elite cultivars. The sequencing of the apple (Velasco et al., 2010; Daccord et al., 2017) and pear (Wu et al., 2013; Chagné et al., 2014) genomes has opened the way to the development of many genomic resources, which also increases the need for accurate tools of gene function analysis in these species. Apple and pear are amenable to genetic transformation since 1989 (James et al., 1989) and 1996 (Mourgues et al., 1996), respectively. Numerous studies have improved genetic engineering tools for apple as well as pear and the number of clonal genotypes amenable to genetic transformation is now about 20 in *Pyrus* and 50 in *Malus* (Malnoy et al., 2008a,b).

Genome editing technologies have tremendously advanced during the last years and they now offer a mean for rational and precise modification of DNA sequences in many plant species. The Clustered Regularly Interspaced Short Palindromic Repeats (CRISPR) nuclease Cas9 efficiently breaks the double strand of DNA at a predefined target site and non-homologous end-joining (NHEJ) permits the recovery of point mutations causing gene knock-out (Belhaj et al., 2013). This targeted mutagenesis technology is rapidly progressing in fruit trees and a number of successful gene knock-outs have been reported in *Citrus* (Jia and Wang, 2014; Jia et al., 2016, 2017; Peng et al., 2017), grape (Ren et al., 2016; Nakajima et al., 2017; Wang X. et al., 2018), kiwifruit (Wang Z. et al., 2018) and cacao (Fister et al., 2018). In apple, a first report indicated successful knock-out of the phytoene desaturase (PDS) gene in the rootstock ‘JM2’ (Nishitani et al., 2016). In this case, the *Streptococcus pyogenes* Cas9 was placed under the control of the CaMV35S promoter and several gRNAs of various lengths (18 or 20 bp) were placed under the control of the AtU6-1 promoter and tested separately. A rate of edition of 31.8% was obtained with clear or partial albino phenotypes. It is thus necessary to improve the methodology of genome editing in apple to reach higher efficiencies and to describe more precisely the complexity of the edition profiles. The only other report of apple gene editing concerns the efficient delivery of CRISPR-Cas9 ribonucleoproteins targeting the apple MLO-7 gene into apple protoplasts (Malnoy et al., 2016). However, no stably edited plants were regenerated from the edited protoplasts. Therefore, the production of T-DNA free edited apple lines is still a challenge. To our knowledge, no report of gene editing via CRISPR-Cas9 has been published so far on pear. The first proof of concept of pear genome editing is still to come.

In the present work our aims were: (i) to obtain high frequencies of gene knock-out of several apple easily scorable genes; (ii) to describe precisely the type of editions in T0 transformants; and (iii) to extend the technology to pear. For this purpose, we chose two target genes. The *PDS* gene disruption results in albino and dwarf phenotypes by impairing chlorophyll, carotenoid and gibberellin biosynthesis (Qin et al., 2007). The *MdPDS* gene is encoded by a single copy gene in the apple genome. The *TFL1* gene is a floral repressor and its silencing leads to accelerate flowering (Shannon and Meeks-Wagner, 1991). Two *TFL1* homologous genes are present in the apple genome and expressed in vegetative tissues (Mimida et al., 2009). *MdTFL1* genes have been silenced through antisense (Kotoda et al., 2006), virus-induced gene silencing (Sasaki et al., 2011) or siRNA (Flachowsky et al., 2012; Weigl et al., 2015). In all cases, early flowering phenotypes were observed. Likewise in pear, expression of an RNAi cassette containing a sequence of the apple *TFL1* led to the inhibition of both *PcTFL-1* and *Pc-TFL-2* and early flowering in the transgenic line (Freiman et al., 2012). We successfully edited *MdPDS* and *MdTFL1.1* genes at high frequencies (>85%) in apple and demonstrated that chimeric biallelic patterns of edition are predominant. The *PcTFL1.1* gene was also edited in pear at a lower frequency.

## Materials and Methods

### Biological Material

Bacterial strains included One Shot^®^ TOP10 Chemically Competent *Escherichia coli* (Thermo Fisher Scientific) for cloning purposes, and *Agrobacterium tumefaciens* EHA105 (Hood et al., 1993) carrying both the binary vector of interest and the ternary plasmid pBBR1MCS-5 with a constitutive *virG* gene (van der Fits et al., 2000) for plant transformation.

The experiments were performed on two genotypes: the apple ‘Gala’ and the pear ‘Conference’. *In vitro* proliferating shoot cultures of the apple ‘Gala’ were micropropagated on Murashige and Skoog (1962) medium supplemented with 0.5 mg/l 6-benzyladenine (BA) and 0.1 mg/l 3-indolebutyric acid (IBA). Cultures of the pear ‘Conference’ were micropropagated as described by Leblay et al. (1991) on a derivative of Lepoivre’s medium supplemented with 0.5 mg/l 6-BA and 0.1 mg/l IBA. All cultures were grown at 22–24° C with a 16:8 h light:dark photoperiod (cool white fluorescent tubes, 40–60 μmol m^-2^ s^-1^) and transferred to fresh medium every 4 weeks.

### Construction of Vectors

**FIGURE 1:**
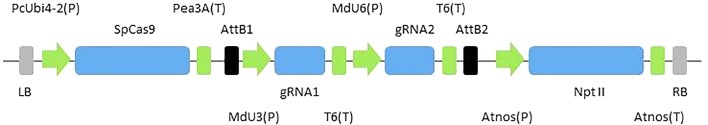
CRISPR-Cas expression construct. The *Cas9* gene from *Streptococcus pyogenes* is driven by PcUbi4-2 promoter (P) from parsley (*Petroselinum crispum*) and transcription is terminated by the Pea3a terminator (T) from pea (*Pisum sativum*). gRNA1 and 2 are, respectively, driven by MdU3 and MdU6 promoters from *Malus domestica* and transcription is terminated by a polyT terminator. Transformants are selected with a *nptII* gene controlled by *nos* promoter and terminator from *Agrobacterium tumefaciens*. AttB1 and 2: sites resulting from the Gateway^®^ LR recombination. LB and RB: T-DNA borders.

Binary vectors CRISPR-PDS and CRISPR-TFL1.1 used in this study were derived from pDE-CAS9 vector (Fauser et al., 2014) (Figure 1). Each construct contained two gRNAs with a different promoter (MdU3 or MdU6) and targeted *MdPDS* (*MD04G0021400*) or *MdTFL1.1* (*AB052994*). First the *bar* resistance cassette in pDE-CAS9 vector was replaced by the *nptII* resistance cassette from pKGW vector (Karimi et al., 2002) by restriction/ligation at HindIII sites to create pDE-CAS9Kr vector. Each gRNA cassette marked out by attB gateway sites was synthesized independently by Integrated DNA Technology, Inc. (San Jose, CA, United States) and then cloned in pDONR207 vector by BP cloning (Gateway system; Thermo Fisher Scientific, MA, United States; Hartley et al., 2000). Then the ‘U6gRNA2’ cassette was placed after the ‘U3gRNA1’ cassette by restriction/ligation at XhoI/PstI sites in the donor vector and SalI/PstI sites in the destination vector, to create pDONR207-U3gRNA1-U6gRNA2 vector. Gateway LR cloning between pDONR2017-U3gRNA1-U6gRNA2 vector and pDE-CAS9Kr vector was then performed to create CRISPR-PDS or CRISPR-TFL1.1. Sequences are given in Supplementary File S1. Primers used to verify the cloning at each step (primers 7 and 8: BP cloning and gRNAs addition, primers 9 and 10: LR cloning) are indicated in Table 1. MdU3 or MdU6 promoters driving gRNAs were, respectively, found upstream MD10G1073100 and MD07G1138500 genes^2^ by BLAST with AtU3 and AtU6 sequences (respectively, found upstream X52629 and X52528; Marshallsay et al., 1990). Sequences are given in Supplementary File S1. Target sequences in gRNA were chosen with CRISPOR software^3^ (Haeussler et al., 2016) using the *Malus domestica* INRA GGDH13 Version 1.1 genome. Sequences are given in Supplementary File S1.

**Table 1:** Primers used in this work.

Sequence	Accessions/reference	Forward primer 5′–3′	Reverse primer 5′–3′
**Cloning control and/or transgenic status control**			
Elongation Factor *Malus domestica*	AJ223969	CTCTTGGTGTCAGGCAAATG (1)	TCAAGGTTGGTGGACCTCTC (2)
23S ribosomal RNA *Agrobacterium tumefaciens*	CP014260.1 gene locus_tag = “AWN88_17620” 1310643..1313449	GTAAGAAGCGAACGCAGGGAACT (3)	GACAATGACTGTTCTACGCGTAA (4)
*nptII* plasmid pK7WG2D	Karimi et al., 2002	ATCGGGAGCGGCGATACCGTA (5)	GAGGCTATTCGGCTATGACTG (6)
Cloning box in pDONR207 plasmid	Thermo Fisher (Invitrogen)	TCGCGTTAACGCTAGCATGGATCTC (7)	GTAACATCAGAGATTTTGAGACAC (8)
gRNAs box in pDEcas9Kr plasmid	This work	AGCTCCCTAGGCCTGTTATC (9)	CTAGGCTGGATCGGAATTATCG (10)
*Cas9* coding sequence in pDEcas9Kr plasmide	Fauser et al., 2014$	TGAGTTGGTGAAGGTGATGGG (11)	TAACGATGTTCACCTGTGGCA (12)
pDEcas9Kr backbone at LB border	Fauser et al., 2014$	TTGCTGCTCCATAACATCAAA (29)	ATACAGGCAGCCCATCAGTC (30)
pDEcas9Kr backbone at RB border	Fauser et al., 2014$	TTTAAAAGGGCGTGAAAAGG (31)	CTTCTCGGAAAACAGCTTGG (32)
**Target sequences cloning and analysis**			
*MdPDS*	MD04G0021400^∗^	AGTGGGCTTGTGTCTCCG (13)	CCGCCTAAAACATCTCTCGC (14)
*MdTFL1*	AB052994	GGGAGGTTTGGGACTAGCAA (15)	TAGACGGCAGCGACAGGAAGA (16)
*PcTFL1*	PCP025869.1	ATGAAAAGAGCATCGGAGC (17)	CTCTGCGCGTTGAAGTAGAC (18)
Cloning box in pJET 1.2 plasmid	Thermo Fisher (Fermentas)	CGACTCACTATAGGGAGAGCGGC (19)	AAGAACATCGATTTTCCATGGCAG (20)
**Off-target genes analysis**			
PEBPMD12	MD12G1023900^∗^	TGAGTTATGAGATGCCGAAGC (21)	TGGGAAACAAAAGTTACAATGG (22)
PEPBMD14-1	MD14G1021100^∗^	ACAAGGATTCCACTTCCAAGC (23)	AGCATTTATACCAGTGCAGGTG (24)
PEPBMD14-2	MD14G1021100^∗^	AAGAGAGGCGCTGAGCTATG (25)	GCACTTTCTCTCTGCGCATT (26)
2-oxoglutarate	MD01G1193900^∗^	GACGGAAAACGCACACATTA (27)	ATGTGCAGAAGAGCCATTCC (28)


**^$^Replacement of DL-Phosphinothricin resistance by kanamycine resistance in this work. ^∗^Available at*https://iris.angers.inra.fr/gddh13*(): primer code*.*

To perform a first proof of concept experiment of pear genome edition, we chose to use the same construct as for apple. However, in one of the two CRISPR-PDS gRNAs designed on the apple genome sequence, the PAM was lost because of a mismatch in the corresponding targets in *PcPDS* and the other gRNA presented also one mismatch with the corresponding target sequence in *PcPDS*, so the construct could not be used on pear. On the contrary, the two CRIPSR-TFL1.1 gRNAs presented one mismatch in position 18 after the PAM for gRNA 1 and no mismatch for gRNA2 in the corresponding targets in *PcTFL1.1*. Therefore the CRISPR-TFL1.1 was chosen to edit the pear genome.

### Plant Transformation

For apple stable transformation, the youngest leaves of 4-week-old microshoots were vacuum-infiltrated in a suspension of *A*. *tumefaciens* at 10^8^ bacteria/ml containing Silwet L-77^®^ (Lehle Seeds, United States) at 0.002%, under -0.9 bar during 1 min. The leaves were then wounded transversely with a scalpel, and placed on apple regeneration medium consisting of MS medium containing 2–5 mg/l thidiazuron (TDZ), 0.5 mg/l IBA and 100 μM acetosyringone, solidified with Phytagel^TM^ (SIGMA, United States) at 3 g/l, in the dark at 22–24°C.

For pear stable transformation, the youngest leaves of 4-week-old microshoots were vacuum-infiltrated in a suspension of *A*. *tumefaciens* at 10^7^ bacteria/ml, under -0.9 bar during 1 min. The leaves were then wounded transversely with a scalpel, and placed on pear regeneration medium (Mourgues et al., 1996) containing 2 mg/l TDZ, 0.5 mg/l naphthalene acetic acid (NAA) and 100 μM acetosyringone, solidified with Phytagel^TM^ (SIGMA, United States) at 3 g/l, in the dark at 22–24°C. For apple and pear, at the end of the co-culture, the leaves were placed on their respective regeneration medium containing 300 mg/l cefotaxime, 150 mg/l timentin and 100 mg/l kanamycin. The explants were kept in the dark and transferred to fresh medium every month for 6 months. Appearance of adventitious buds was monitored for a period of 6 months. All regenerated buds were micropropagated on the same medium as their mother plants, with the addition of 300 mg/l cefotaxime, 150 mg/l timentin, and 100 mg/l kanamycin.

For apple transient transformation, the protocol was modified as follows: the inoculum was a mix of the strain with a CRISPR construct and a strain carrying the gene coding the p19 protein of tomato bushy stunt virus as a suppresser of gene silencing (Voinnet et al., 2003), respectively, at 5 × 10^8^ bacteria/ml and 2.5 × 10^8^ bacteria/ml, supplemented with 0.002% Silwet L-77^®^. After agroinfiltration, the leaves were placed on regeneration medium containing 300 mg/l cefotaxime, 150 mg/l timentin, and 100 mg/l kanamycin for 1 week. Leaves were then transferred to the same medium without kanamycin. The explants were kept in the dark and transferred to fresh medium without kanamycin every month for 3 months. All regenerated buds were micropropagated on the same medium as their mother plants, with the addition of 300 mg/l cefotaxime, 150 mg/l timentin, without kanamycin.

### Presence of Transgenes

Presence of transgenes and absence of contaminating agrobacteria were monitored by PCR. Genomic DNA of apple and pear leaves was extracted as described in Fulton et al. (1995). The primers used for the detection of (i) the gRNAs cassette (9 and 10), (ii) the *Cas9* coding sequence (11 and 12), (iii) *A. tumefaciens* presence (3 and 4), (iv) *nptII* gene (5 and 6) and (v) elongation factor 1α (*EF1*α) coding gene as a marker of plant DNA suitability for PCR (1 and 2) are listed in Table 1. Amplifications were performed using GoTaq^®^ Flexi DNA Polymerase (Promega, Madison, WI, United States) according to the manufacturer’s recommendations. The PCR reaction conditions were identical for the five genes: 95°C for 5 min, followed by 35 cycles at 95°C for 30 s, 58°C for 45 s, 72°C for 1 min, with a final extension at 72°C for 5 min. The PCR products were separated on a 1.5% agarose gel. Absence of plasmid backbone was also monitored in putative T-DNA free edited lines. Primers pairs used on either sides of the T-DNA (left: 29 and 30; right: 31 and 32) are listed in Table 1. The PCR reaction conditions were as above but the hybridization at 53°C.

### Detection of Mutations

For mutation analysis of each target region, primer pairs were designed based on apple *MdPDS* (primers 13 and 14) and *MdTFL1.1* (primers 15 and 16) genomic sequences from the apple ‘Golden Delicious’ genome or on the *PcTFl1.1* (primers 17 and 18) genomic sequence from *Pyrus communis* (PCP025869.1). These primers amplified a DNA fragment of approximately 600–750 bp for *MdPDS* gene and approximately 2 kb for *TFL1.1* (*Md* and *Pc*) genes surrounding each target. The wild type apple *MdPDS* and *MdTFL1.1* gene fragments were amplified by PCR using genomic DNA from ‘Gala’. In the same way, the wild type pear *PcTFL1.1* gene fragments were amplified by PCR using genomic DNA from ‘Conference.’ All primer pairs were described in Table 1. The PCR reaction conditions for *MdPDS* gene were as described in part 2.4. The PCR reactions for the *TFL1.1* gene in apple and pear were performed with the following conditions: 95°C for 5 min, followed by 40 cycles at 95°C for 30 s, 58.5°C for 45 s, 72°C for 3 min, with a final extension at 72°C for 5 min. A touch-down PCR program (Marchand et al., 2003) (initial annealing temperature of 59°C, decreasing by 0.5°C per cycle down to 56°C) was used on a few recalcitrant pear and apple *TFL1.1* samples using Q5^®^ High-Fidelity DNA polymerase (New England Biolabs, MA, United States) following by gel purification to isolate the right amplicon.

Blunt and ligation reactions were performed on amplification products using the Sticky-End cloning protocol from CloneJET PCR Cloning Kit (Thermo Scientific, Waltham, MA, United States) according to the manufacturer’s recommendations. Bacterial transformation was performed in One Shot^TM^ TOP10 Chemically Competent *E. coli* and spread on LB plates with 50 μg/ml ampicillin. *MdPDS* and *TFL1.1* insertion colonies were checked by PCR (primers 19 and 20) using GoTaq^®^ Flexi DNA Polymerase with pJET 1.2 primers (Table 1) using conditions described in part 2.4. The bacteria containing putative edited sequences were directly sent for sequencing. All sequencing results were compared with the reference sequence of the wild type apple *MdPDS* (*MD04G0021400*) or *TFL1.1* (*AB052994*) genes and pear (PCP025869.1) *TFL1.1* gene by alignment in MultAlin software (Corpet, 1988).

Putative off-target genes for gRNAs in our constructions were obtained thanks to CRISPOR software^4^ (Haeussler et al., 2016). For mutation analyses in putative off-target genes, strategy was as before: genes primers pairs (21 and 22, 23 and 24, 25 and 26, 27 and 28; Table 1) were designed based on genomic sequences from the apple ‘Golden Delicious’ genome (Daccord et al., 2017). They amplified a DNA fragment between 201 and 475 bp surrounding each putative target. PCR conditions were as described in part 2.4 and subsequent cloning and sequencing were as described just above.

## Results

### Production of Apple and Pear Transgenic Lines

In total, five stable transformation experiments were performed and resulted in variable rates of transformation (Table 2). For the CRISPR-PDS construct, 57 ‘Gala’ kanamycin resistant lines were produced in a single experiment whereas three experiments were needed to produce 30 ‘Gala’ kanamycin resistant lines with the CRISPR-TFLl.1 construct. For pear, a very high rate of transformation was achieved (23%) and 100 ‘Conference’ kanamycin resistant lines were produced with the CRIPSR-TFL1.1 construct. Only 54 lines were further studied.

**Table 2:** Production of transgenic lines.

Genotype	Binary vector	Transformation experiment	Number of leaves inoculated	Number buds regenerated	Number transgenic lines	Rate of transformation
‘Gala’	CRISPR-PDS	N° 295	400	126	57	14.25%
‘Gala’	CRISPR-TFL1.1	N° 292	400	23	3	0.75%
		N° 296	419	41	23	5.49%
		N° 298	400	16	4	1.00%
‘Conference’	CRISPR-TFL1.1	N° 71	412	117	100	24.27%


The ‘Gala’ kanamycin resistant-lines with the CRISPR-PDS construct which survived after 1 year of micropropagation were analyzed by PCR and *EF1*α gene was used as a marker of plant DNA suitability for PCR. Plasmid DNA extracted from *A. tumefaciens* strain containing the CRISPR-PDS construct and genomic DNAs extracted from a non-transgenic ‘Gala’ were used as controls. Amplification with UF/B1R primers showed that all the tested lines were free from *A. tumefaciens* contamination. Amplification with *nptII* primers confirmed that the 41 tested lines were true transgenic lines carrying the selectable marker gene. Amplification with primers for the gRNAs presence and for the Cas9 coding sequence showed that 39 of the 41 lines had integrated the full CRISPR-PDS construct. Two additional albino lines did not integrate the gRNAs box and the Cas9 coding gene. For the transgenic lines with the CRISPR-TFL1.1 construct, we decided to study only a sample of five early flowering lines per species. All of them had integrated the full CRISPR-TFL1.1 construct and were free from *A. tumefaciens*.

### Phenotypic Analysis of Transgenic Lines

**FIGURE 2:**
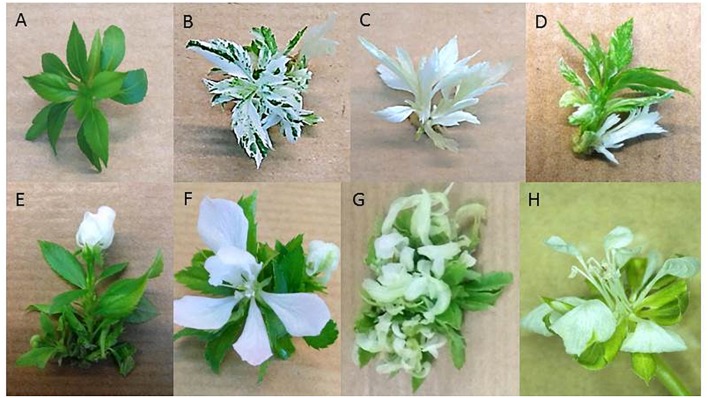
Transgenic lines phenotypes. Phenotypes of ‘Gala’ transgenic lines containing the CRISPR-PDS construct: **(A)** pure green, **(B)** variegated, **(C)** pure white, **(D)** variegated line undergoing chimera dissociation through adventitious bud formation. Phenotypes of ‘Gala’ transgenic lines containing the CRISPR-TFL1 construct: **(E)** flower bud formation in apical position, **(F)** opened flower with all floral organs present, **(G)** loss of vegetative growth after 3 months of continuous flowering. Phenotype of ‘Conference’ transgenic line containing the CRISPR-TFL1construct: **(H)** opened flower with all floral organs present.

The expected phenotype of knock-out mutants of the *PDS* gene is dwarf and albinos. After the first subculture of the transgenic buds expressing the CRISPR-PDS construct on micropropagation medium, their original phenotype was recorded. At this early stage, 15 lines (26%) appeared pure white and 6 lines (10%) pure green. All the other lines (64%) showed various mixtures of white, green and variegated leaf phenotypes indicating a high level of chimerism (Figure 2A–C). All the transgenic lines were micropropagated over a period of 1 year and white sectors were selected at each subculture (Figure 2D). This led to the progressive disappearance of variegated phenotypes (only one line) and a majority of pure white (48 lines) or green (9 lines) remained. A rate of 84% of edition (48 out of 57 lines) was finally recorded.

**FIGURE 3:**
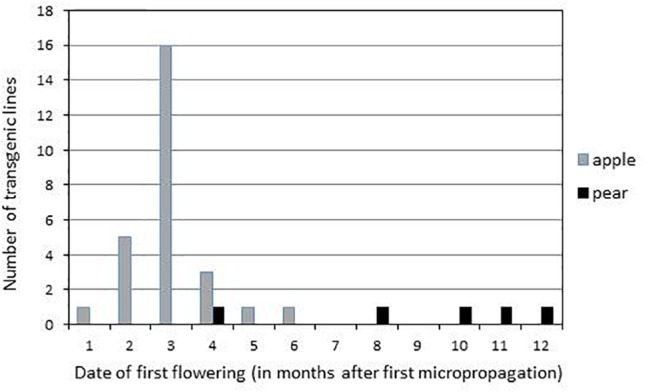
Date of first flowering of transgenic lines expressing the CRISPR-TFL1.1 construct.

Flower buds appeared on terminal position on ‘Gala’ and ‘Conference’ transgenic lines expressing the CRISPR-TFL1.1 construct and the opened flowers showed the presence of all floral organs, often in irregular numbers (Figure 2). Flowering of ‘Gala’ transgenic lines occurred between one and 6 months after the beginning of their micropropagation (Figure 2E–H). Similarly to the albino phenotype, the flowering phenotype appeared progressively, first on one then on several shoots of each transgenic line. When the majority of the apical meristems of a single line turned floral, its micropropagation became very difficult due to the cessation of vegetative growth (Figure 2G). Most ‘Gala’ transgenic lines flowered after 3 months, and after 6 months 27 out of 30 lines had flowered, indicating a rate of 90% of edition. On the contrary, flowering of ‘Conference’ transgenic lines appeared between 4 and 12 months after the start of their micropropagation (Figure 3), and was limited to 5 lines out of 54, indicating a rate of edition of 9%.

### Characterization of Targeted Mutation of *MdPDS* and *TFL1.1* Genes

**FIGURE 4:**
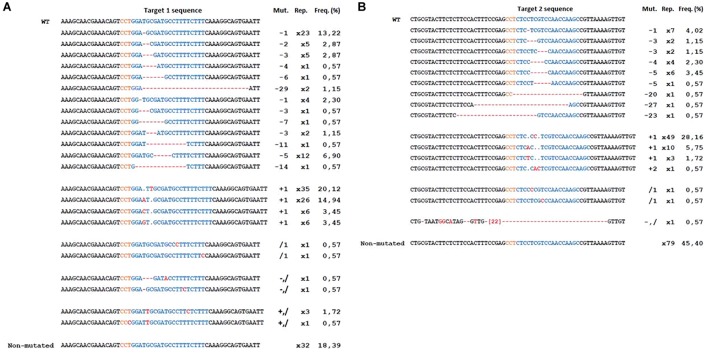
Mutations in the apple *MdPDS* gene induced by CRISPR/Cas9. **(A)** Results for target 1, **(B)** results for target 2. Alignment was done in comparison to the wild type (WT). In color blue, the targeted sequence. This synthesis excludes two particular cases described in Figure 5 but included in the total for the frequency calculation. In orange, the PAM sequence. Mut: type of mutation with “–” for deletion, “+” for addition, “/” for substitution. Rep, number of occurrences of each indel. Freq, (%) frequencies calculated in relation to the number of occurrences of each indel among the total number of bacterial clones sequenced (174).

**FIGURE 5:**
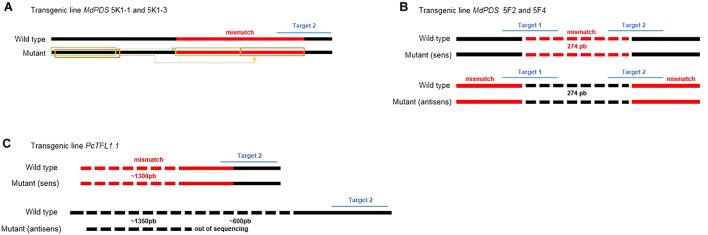
Schemes of particular edition profiles involving large scale mutations. **(A)** Mutation on target 2 of the *MdPDS* gene, observed on 2 bacterial clones from one edited line (MdPDS 5K1-1 and 5K1-3): a large mismatch (57 bp) concerns a part of target 2 resulting from a substitution of copies of two fragments upstream of the sequence. **(B)** Mutation concerning the two targets of the *MdPDS* gene, observed on 2 bacterial clones from one edited line (MdPDS 5F2 and 5F4): the large mismatch (274 bp) between the two targets results from the sequence reversal after Cas9 cutting and repair by the NHEJ system. **(C)** Mutation on target 2 of the *PcTFL1.1* gene, observed on the 8 bacterial clones from the two edited lines PcTFL1.1 71AJ and 71CI: substitution of a reverse fragment from a locus upstream of the gene. Sequencing being limited in length, the mutation is not completely described and the inversion could concern a fragment longer than 1300 bp.

**FIGURE 6:**
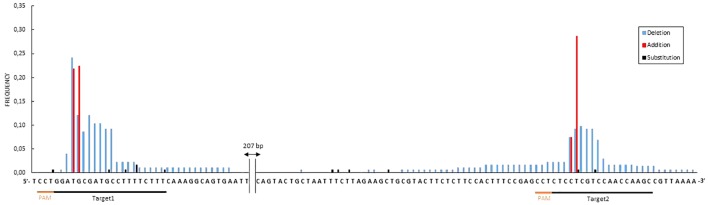
Frequencies of nucleotide editions of MdPDS targets according to indel positions. Frequencies were calculated in relation to the total number of edited MdPDS clones (174). Particular cases (alleles 5F2, 5F4 and 5K1-1, 5K1-3) are not presented. The distance between the end of the 1st target and the beginning of the 2nd is 272 bp.

In total, 41 transgenic lines (subsequently named ‘lines’) with the CRISPR-PDS construct were analyzed including 37 albino and 4 green lines. For the majority of these lines, four bacterial clones (subsequently named ‘clones’) per transgenic line were sequenced, each containing an allele of the target sequence putatively edited. For two lines, only three clones could be obtained. In addition, for three lines 8 clones were sequenced in order to further explore the variability of edition profiles. A summary of the edition profiles is given in Table 3. For the *MdPDS* gene, a majority of edited lines were chimeric (87.8%). Among these chimeric lines, all albino lines presented at least one biallelic cell line and 8 transgenic lines presented at least one heterozygous cell line. Three of the four green lines were non-mutated and the last green line presented an edition on one clone out of four only. Among the 174 bacterial clones sequenced, both targets were simultaneously mutated in 51.1% of cases, target 1 was more often mutated alone (30.5%) than target 2 (3.5%). Small indels located in the target sequence were the most frequent outcome with 78.2% for target 1 and 50% for target 2. A great variability of types of indels was observed (Figure 4): 24 types of indels for target 1 and 17 for target 2. For target 1, 48.3% of the clones were represented by three specific indels: addition of a thymine at the 5th nucleotide after the PAM, addition of an adenine at the 4th nucleotide after the PAM and deletion of a thymine at the 4th nucleotide after the PAM. For target 2, 28.2% of the clones presented an addition of a cytosine at the 5th nucleotide after the PAM. Substitution indels were located more randomly in target sequence and frequently combined with deletions or additions. Larger deletions ranging from 11 to 29 bp were less frequent. Two particular cases involving reshuffling of large sequence fragments are detailed in Figure 5, the first case (A) resulted from a large substitution by a double copy of an upstream sequence, the second case (B) resulted from an inversion of the sequence between the two targets. Figure 6 presents the frequencies of the three types of indels according to their position at the target site. The majority of deletions (96.7% for target 1, 84.6% for target 2) were located only in targets. All additions were located on the 4th and the 5th nucleotides after PAM for both targets.

**Table 3:** Summary of edition profiles of transgenic lines.

	CRISPR-PDS apple	CRISPR-TFL1.1 apple	CRISPR TFL1.1 pear
Total lines analyzed (#)	41	5	5
Homozygous^1^ lines (#)	0	0	1
Heterozygous^2^ lines (#)	8	0	0
Biallelic^3^ lines (#)	37	5	4
Chimeric lines	36	5	4
Non-edited lines	3	0	0
Total clones sequenced (#)	174	20	20
Target 1 mutated alone (#)	53	0	0
Target 2 mutated alone (#)	6	0	0
Targets 1 and 2 mutated (#)	89	20	20
Non-mutated clones (#)	26	0	0


*^*1*^Homozygous = both alleles are mutated and present the same mutation.*

*^*2*^Heterozygous = only one allele is mutated. ^*3*^Biallelic = both alleles are mutated but mutations are not identical. ^*4*^Chimeric = presence of different edited cell line profiles.*

**FIGURE 7:**
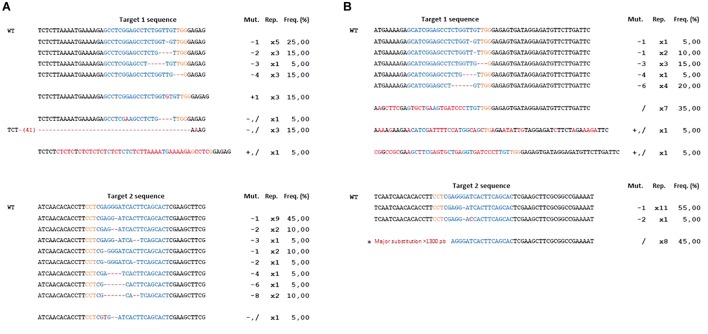
Mutations in the apple *MdTFL1.1* **(A)** and pear *PcTFL1.1* **(B)** genes induced by CRISPR/Cas9. Particular case (^∗^) is described in Figure 5. In orange, the PAM sequence. Mut: type of mutation with “–” for deletion, “+” for addition, “/” for substitution. Rep, number of repetitions of each indel. Freq, (%) frequencies calculated in relation to the number of repetition of each indel among the total number of clones sequenced (20 for both species).

A sample of five apple and five pear early flowering transgenic lines with the CRISPR-TFL1.1 construct were analyzed and four bacterial clones were sequenced per line. As for the CRISPR-PDS construct, results (Table 3) indicated a majority of chimeric biallelic profiles. However, one pear transgenic line showed an homozygous non-chimeric profile. The two targets were simultaneously edited in all apple and pear lines with the CRISPR-TFL1.1 construct, despite the presence of one mismatch between the gRNA1 and the target in pear. All edited lines for *MdTFL1.1* and *PcTFL1.1* analyzed are described in Figure 7. For both genes, a majority of alleles presented a deletion of one or more bases at the target sites. On target 1, major editions, including substitutions and additions were observed for 9 among 20 edited alleles. On target 2, 8 edited alleles presented a major reshuffling substitution schematized in Figure 5.

### Analysis of Potential Off-Target Mutations

Potential off-target sequences were identified using the CRISPOR software. We found no off-target genes for gRNA2 in CRISPR-PDS construct and two for gRNA1 with three mismatches each. In CRISPR-TFL1.1 construct, we found two putative off-target genes for each gRNA. gRNA1 could target MD12G1023900 and MD14G1021100 with, respectively, zero and three mismatches and gRNA2 could target MD14G1021100 and MD01G1193900 with three mismatches each (Table 4). Given the greater probability to affect off-target genes with CRISPR-TFL1.1 construct than with CRISPR-PDS construct, we analyzed only putative off-target genes of CRISPR-TFL1.1 construct.

**Table 4:** Analysis of the off-target gene with 0 mismatch for CRISPR-TFL1.1 gRNA1.

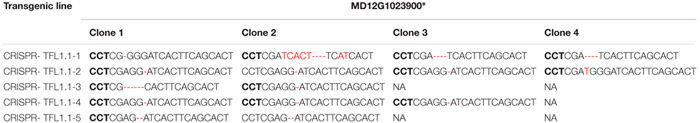

**PAM is in bold, editions are in red, ‘-’: deletions. NA, non-available. ^∗^Available at*https://iris.angers.inra.fr/gddh13.*

Two to four bacterial clones were sequenced for each off-target gene in five different transgenic lines (CRISPR-TFL1.1-1 to 5). We failed to design specific primers to amplify the putative target of gRNA2 in MD14G1021100. We found no mutations in putative off-target genes with three mismatches but, in each transgenic line, we found editions of MD12G1023900, targeted by gRNA1 without mismatch (Table 4).

### Production of T-DNA Free Edited Lines

**FIGURE 8:**
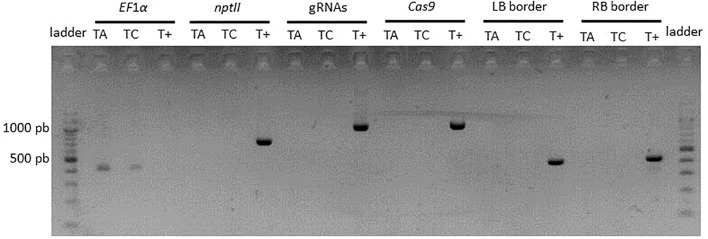
PCR analysis of T-DNA free edited lines. TA, T-DNA free edited line A. TC, T-DNA free edited line C. T+, binary vector CRISPR-TFL1.1 as a control.

In order to evaluate the feasibility of production of T-DNA-free edited lines, a transient transformation with the CRISPR-PDS construct was performed on apple. The protocol was adapted to increase the frequency of transient expression by (i) a higher concentration of inoculum, (ii) co-inoculation with an *A. tumefaciens* strain carrying an inhibitor of silencing, (iii) kanamycin selection applied only during the period of T-DNA transient expression (1 week after the end of the co-culture). In total, 229 apple leaves were inoculated. A very high rate of regeneration was observed (71%). Among 747 regenerated buds, three albino buds were detected (0.4% mutation efficiency). PCR analysis indicated for two of them the absence of integration of *Cas9* or *nptII* genes, gRNAs and left and right backbone sequences (Figure 8). Sequencing of four clones per edited line indicated that the two T-DNA free lines were homozygous and mutated only on target 1. One line showed a single deletion at the fourth base after the PAM and the other line presented a more complex mutation with three insertions and four substitutions. These two lines can be considered as T-DNA free edited lines.

## Discussion

### High Efficiencies of Editing Can Be Obtained in Apple by the Simultaneous Use of Two gRNAS Driven by Apple U3 and U6 Promoters

The present study demonstrates successful CRISPR-Cas9-mediated targeted edition of two different genes in apple, with very high rates of edition (84–90%) in the first generation after transformation (T0 plants). These ratios are higher than comparable ratios of other perennial crop: 51.7% of edited T0 plants in poplar (Fan et al., 2015), 30.6% in grape (Wang X. et al., 2018), 34.5% in orange (Peng et al., 2017). The only other report of CRISPR-Cas9 edition of the *MdPDS* gene in regenerated apple plants indicated a rate of edition of 31.8% in the rootstock ‘JM2’ (Nishitani et al., 2016). The difference of genotype could explain in part the difference of edition efficiency. But other differences in the construct design must be taken into account. First, Nishitani et al. (2016) used a *Cas9* fused to *GFBSD2* (GFP fused to the N-terminus of blasticidin S deaminase) under the control of the CaMV35S promoter. This fusion protein may be expressed less efficiently than the simple Cas9 [codon-optimized for *Arabidopsis thaliana* by Fauser et al. (2014)] placed under the control of the parsley ubiquitin promoter, in our study. Secondly, we used two gRNAs simultaneously and placed them under the control of the apple U3 and U6 promoters, whereas Nishitani et al. (2016) used several gRNAs separately under the control of the *A. thaliana* U6 promoter. This strategy probably increased the probability of edition of the target gene.

### Pear *TFL1.1* Gene Can Be Edited Using gRNAs Designed on the Apple Genome

Our study also reports for the first time the CRISPR-Cas9- mediated targeted mutation of a pear gene (*PcTFL1.1*) with a rate of edition of 9% of the observed T0 plants. To perform this first proof of concept experiment of pear genome edition, we chose to use the same construct as for apple edition of *MdTFL1.1* gene. However, the CRIPSR-TFL1.1 gRNA1 designed on the apple genome sequence presented one mismatch in position 18 after the PAM with the corresponding target sequence in pear *PcTFL1.1*. The presence of this mismatch could explain the lower rate of mutated phenotype observed in pear. It is also possible, that in pear, the knockout of both *TFL1* genes (*PcTFL1.1* and *PcTFL1.2*) is necessary to release totally the floral repression. The only previous experiment to silence pear *TFL1* used an RNAi construct based on *MdTFL1.1* which silenced both pear *TFL1* genes and led to an early flowering phenotype (Freiman et al., 2012). Yet, in our study, both gRNAs in the CRIPSR-TFL1.1 construct presented three mismatches with the corresponding target sequences in pear *PcTFL1.2*. Thus, in light of our results on off-target genes, we assume that *PcTFL1.2* was not edited. On the contrary, targeting *MdTFL1.1* alone and not other *TFL1*/*CEN*-like genes (*MdTFL1a*, *MdCENa*, and *MdCENb*; Mimida et al., 2009) appeared to be sufficient to get the early flowering phenotype. Indeed CRIPSR-TFL1.1 gRNA1 and gRNA2 showed three or more mismatches with the corresponding target sequences in those *TFL1*/*CEN*-like genes, which exclude their edition in our study.

### Complex Edition Profiles and Chimerism Are Frequent in Apple and Pear Edited Lines

Phenotypic and molecular analyses of the PDS-edited apple lines produced in our study clearly indicated that the Cas9 protein is acting progressively during the whole period of stable transgenic bud regeneration (about 6 months). As already described by Nishitani et al. (2016), a majority of transgenic CRISPR-PDS lines initially appeared as a mixture of white, green and variegated sectors, indicating a high level of chimerism between edited and non-edited tissues in the same plant. Similarly, the flowering phenotype appeared very progressively on apple and pear CRISPR-TFL1.1 lines. The sequencing analyses were performed about 1 year after the regeneration of the transgenic buds, and a constant selection for the mutated phenotypes (albino or flowering) was applied during this period. Therefore most of the non-edited tissues were probably eliminated at the time of the molecular analyses. The sequencing of more than 214 bacterial clones revealed a minority of non-mutated alleles (12%). In the majority of lines (88%), more than two mutated allele sequences were detected, indicating chimerism between different edited cell-lines in the analyzed tissues. Up to six different alleles were observed in one of the lines for which eight clones were sequenced. Furthermore, bi-allelic mutants were the most frequent outcome and only one putatively mono-allelic homozygous line was observed among the 51 independent edited lines analyzed. Multiple mutated sequences and frequent biallelic mutations were also observed in grape (Nakajima et al., 2017; Wang X. et al., 2018), orange (Peng et al., 2017) or tomato (D’Ambrosio et al., 2018) T0 plants.

A clear difference of efficiency between the two gRNAs was observed in *MdPDS*-edited lines but not in *MdTFL1.1* or *PcTFL1.1*-edited lines. Most of the edition profiles observed in our study were small additions or deletions at the targets sites, as a consequence of repair through NHEJ after gRNA-directed Cas9 cleavage. This is in agreement with the majority of reports which indicate that about half of CRISPR-Cas9-induced mutations are single-base insertions and the rest small deletions (Ma et al., 2016). It has been reported that multiple gRNAs targeting close targets in one gene can result in a large deletion in poplar (Fan et al., 2015). Similarly, paired-sgRNA vectors led to large fragment deletions in kiwifruit (Wang X. et al., 2018; Wang Z. et al., 2018). Despite of the use of two gRNAs, no deletions between the two targets were observed in our study. The only case of large mutation involving the two targets was the inversion of the fragment between the two targets observed in one edited line. In the previous report on apple using CRISPR-Cas9 to target *MdPDS* (Nishitani et al., 2016), only insertion or deletions (+1 to -8 bases) were obtained. This is probably due to the use of a single gRNA.

### Occurrence of Off-Target Edition Depends on the Number of Mismatches in the gRNAs

It is well known that Cas9 can act on non-selective regions of genomic DNA known as “off-target” sites (Kadam et al., 2018). A high homology between the desired target and other zones in the genome increases the risk of off-target activity. Our results on apple confirmed that when complete homology exists between a gRNA and a non-target sequence, this off-target sequence was systematically edited. Our results on pear showed that in case of one mismatch distal from the PAM, the target sequence is also edited. On the contrary, potential off-target sequences containing three mismatches with the gRNA were never edited in apple.

### Transient Expression Permits the Production of T-DNA Free Apple Edited Lines at Low Frequency

Using a modified protocol in order to favor transient expression of *Cas9*, we have been able to produce two lines mutated for *MdPDS* without integration of T-DNA elements or backbone sequences. To our knowledge, this is the first case of production of T-DNA free edited plants in a woody fruit species. The fact that these two lines present a single homozygous mutation at the target 1 site indicates a very early activity of Cas9 at the beginning of the regeneration process. However, the overall efficiency of this transient system remains very low (0.4% of edited lines). This is lower than the mutation frequencies obtained after agroinfiltration of CRISPR constructs targeting PDS gene in *Nicotiana benthamiana* leaves (Li et al., 2013; Nekrasov et al., 2013; Chen et al., 2018). The only other attempt to produce apple T-DNA free edited lines was through direct delivery of CRISPR-Cas9 ribonucleoproteins to apple protoplasts (Malnoy et al., 2016). Efficient edition of two target genes (*MLO-7* and *DIPM-1*) was observed in the protoplasts after PEG treatment, but no plants were regenerated.

## Conclusion

Our overall results indicate that CRISPR-Cas9-mediated knockout of targeted genes is very efficient in apple and possible in pear. Careful design of gRNAs without mismatch with the target gene should increase the frequency of pear edition. The high proportion of biallelic mutants obtained in our study is a good indication that CRISPR-Cas9-mediated knock-out is an efficient strategy for loss of function experiments in apple or pear. However, occurrence of chimerism in T0 plants is of particular concern for the genome editing of these trees which cannot be crossed to segregate the chimera without losing the genotype of the edited variety. Strategies to separate chimeras before analysis of the edited lines should be developed. Adventitious regeneration already proved to be efficient for chimeral release in naturally variegated cultivars of pear (Abu-Quaoud et al., 1990). Finally, our study demonstrates that the production of T-DNA free edited apple lines is feasible. Various strategies such as biolistics or viral delivery could be tested to increase the efficiency of recovery of these lines. In conclusion, the CRISPR-Cas9 system presented here now allows the establishment of a robust and precise genomic edition platform in apple and pear.

## Author Contributions

AC and EV performed part of the experiments, analyzed the data, and revised the manuscript. ND and AR performed part of the experiments and revised the manuscript. AP performed part of the experiments, analyzed the data, and drafted the manuscript. EC designed the study and drafted the manuscript. All authors read and approved the final manuscript.

## Conflict of Interest Statement

The authors declare that the research was conducted in the absence of any commercial or financial relationships that could be construed as a potential conflict of interest.

## Footnotes

1 http://www.fao.org/

2 https://iris.angers.inra.fr/gddh13

3 crispor.tefor.net/

4 crispor.tefor.net/
