# Supplementary material for: Efficient Targeted Mutagenesis in Apple and First Time Edition of Pear Using the CRISPR-Cas9 System
Source: Front Plant Sci. 2019 Feb 6;10:40. doi: 10.3389/fpls.2019.00040 (PMC6373458; doi:10.3389/fpls.2019.00040)
Supplement: Supplementary file 1 [file Table_1.DOCX]

**File S1: Sequences used to build vectors CRISPR-PDS and CRISPR-TFL1.1.** Bold: promoter, red: target sequence, blue: guide RNA scaffold, italic: termination sequence, lowercase: Gateway attB sites, other: restriction sites.

>AtU3

**acgaccttacttgaacaggatctgttctataggctcgtacctctgtttccttgatttctcaagagacaggcccttaaccctggttgatgaaccatgaccgtgcggctagagcgtgattgacggctacgatcgtcctcggacgcatccggtgctgtagaggatcgttactcggctcggtttctaccttgccggggtggtcgcacggcggtctgacaggtccctttccttttttcttttttttgccataaacttaaatttgtatatcgatcattgtagatattgaaaacctagaacaaaccaacatccatgtgaatgtctttcatgactgatttagagataattcttgaattttggaactagaatctataatgagcct**

>AtU6

**gtcccttcggggacatccgataaaattggaacgatacagagaagattagcatggcccctgcgcaaggatgacacgcataaatcgagaaatggtccaaattttttttgcaaaattttccagatcgatttcttcttcctctgttcttcggcgttcaatttctgggtttttctcttcgttttctgtaactgaaacctaaaatttgacctaaaaaaaatctcaaataatatgattcagtggttttgtacttttcagttagttgagttttgcagttccgatgagataaaccaataactttgcttagatctaattcattccgttacacctctgatggagatggaaggttcttaataatgatgccattttttgggtaataattttgaattagaatcaagggtataagattcataattaacatcacttaagcaaagttcgtaatatacgaccacaggatataatttttgaattc**

>U3gRNA1_PDS

ggggacaagtttgtacaaaaaagcaggcttc**GAGCTCGAATTCAAAGTAGAACTAGAATGTTTAAATAAAAAGGTTGCTAGTATTCTCTTAACATTAATTAAGCATACGTCCAACTTGTTTATCTTTTAACCCAAAAACGTATCCCCAATTAACTCACACAGGTTTTTCTGTATAAATTTTAGAGATAGTAGTTTCGGCTCCCAAATCTATTGAAAACTAAAGGAACAAAAAACAGAAGAGAGAAGAGGAGCGAGAGCGCTCTTAGCTGTTAAAACGAAACATCTCATTTCTTGTCCCACATCGACCGTTTCCAGATTACTAAAGCTGCTTATATGCCTAAACAATGACCAACTGTTC**aAAGAAAAGGCATCGCATCCGTTTTAGAGCTAGAAATAGCAAGTTAAAATAAGGCTAGTCCGTTATCAACTTGAAAAAGTGGCACCGAGTCGGTGC*TTTTTTT*GAGCTCGTCgacccagctttcttgtacaaagtggtcccc

>U6gRNA2_PDS

ggggacaagtttgtacaaaaaagcaggcttc**GAGCTCCTCGAGTTTTGGATTACCATTCTTAAAATTTGAAGCTGTGAATTTGTGTCAACCTATACTTGTACAACATATGACATGCCATATTGTTTCTGTAAGTGGAGTTGTCAACCTGCCCGTCTAGCTCAGTTGGTAGAGCGCAAGGCTCTTAACCTTGTGGTCGTGGGTTCGAGCCCCACGGTGGGCGCTGCTTTTTATTTTTAACTTTTTTTAATCGACAAATACGCTGCGTTTTACAAAAAGTGGTGGAGGAGGTGTCCCACATCGAGCAAACGCAGTGGTATTAATTGCTTTATATTCAATTAGACTGCAAAAAGTGTT**gCTTGGTTGGACGAGGAGGTTTTAGAGCTAGAAATAGCAAGTTAAAATAAGGCTAGTCCGTTATCAACTTGAAAAAGTGGCACCGAGTCGGTGC*TTTTTTT*GAGCTCGAATTCgacccagctttcttgtacaaagtggtcccc

>U3gRNA1_TFL1.1

ggggacaagtttgtacaaaaaagcaggcttcG**AGCTCGAATTCAAAGTAGAACTAGAATGTTTAAATAAAAAGGTTGCTAGTATTCTCTTAACATTAATTAAGCATACGTCCAACTTGTTTATCTTTTAACCCAAAAACGTATCCCCAATTAACTCACACAGGTTTTTCTGTATAAATTTTAGAGATAGTAGTTTCGGCTCCCAAATCTATTGAAAACTAAAGGAACAAAAAACAGAAGAGAGAAGAGGAGCGAGAGCGCTCTTAGCTGTTAAAACGAAACATCTCATTTCTTGTCCCACATCGACCGTTTCCAGATTACTAAAGCTGCTTATATGCCTAAACAATGACCAACTGTTC**aGTGCTGAAGTGATCCCTCGGTTTTAGAGCTAGAAATAGCAAGTTAAAATAAGGCTAGTCCGTTATCAACTTGAAAAAGTGGCACCGAGTCGGTGC*TTTTTTT*GAGCTCGTCgacccagctttcttgtacaaagtggtcccc

>U6gRNA2_TFL1.1

ggggacaagtttgtacaaaaaagcaggcttc**GAGCTCCTCGAGTTTTGGATTACCATTCTTAAAATTTGAAGCTGTGAATTTGTGTCAACCTATACTTGTACAACATATGACATGCCATATTGTTTCTGTAAGTGGAGTTGTCAACCTGCCCGTCTAGCTCAGTTGGTAGAGCGCAAGGCTCTTAACCTTGTGGTCGTGGGTTCGAGCCCCACGGTGGGCGCTGCTTTTTATTTTTAACTTTTTTTAATCGACAAATACGCTGCGTTTTACAAAAAGTGGTGGAGGAGGTGTCCCACATCGAGCAAACGCAGTGGTATTAATTGCTTTATATTCAATTAGACTGCAAAAAGTGTT**gCCTCGGAGCCTCTGGTTGTGTTTTAGAGCTAGAAATAGCAAGTTAAAATAAGGCTAGTCCGTTATCAACTTGAAAAAGTGGCACCGAGTCGGTGC*TTTTTTT*GAGCTCGAATTCgacccagctttcttgtacaaagtggtcccc
